# Increasing Epstein-Barr virus infection in Chinese children: A single institutional based retrospective study.

**DOI:** 10.12688/f1000research.15544.2

**Published:** 2019-02-15

**Authors:** Kiran Devkota, Maio He, Meng Yi Liu, Yan Li, You Wei Zhang

**Affiliations:** 1 Department of Pediatrics, Renmin Hospital, Hubei University of Medicine, Shiyan, Hubei, 44200, China

**Keywords:** Infectious mononucleosis; Prevalence; EBV DNA; Epstein–Barr virus

## Abstract

The Epstein-Barr virus (EBV) is a common virus in humans and the most common causative agent of Infectious Mononucleosis. EBV primary infection has recently risen in some countries and children below 2 years of age are highly susceptible. The clinical manifestations in children with EB virus infection involve multiple systems, causing severe illness, meaning attention should be paid during diagnosis and treatment.

**Objective:**  This single institution based retrospective study was carried out with the aim of estimating the overall prevalence of EBV infection and identifying high-risk age group among children.

**Methods:** This study include total 253 patients under 15 years of age found to be  positive for EBV DNA by serum PCR who were admitted to the Pediatrics Department of Renmin Hospital,(Shiyan, China) during a 4-year period from 2014 to 2017. Patients were divided into three groups; 0-<4years, 4-<6years and 6-<15years. We then calculated the percentage and prevalence of EBV DNA-positive cases.

**Results:** The yearly EBV prevalence rate was 4.99 per 1000 admissions in 2014, 6.97 per 1000 admissions in 2015, 10.42 per 1000 admissions in 2016, and 12.16 per 1000 admissions in 2017. Out of 253 EBV-positive cases, those under 4 years had the highest rate of EBV infection (74.7%). The rate drops to 11.06% in the 4-6 years group, and was 14.22% in the 6-15 years group. Those between 6 months and 1 year are those at the highest risk.

**Conclusion:** The rate of hospital admission of children due to EBV infection is increasing day by day. Children under 4 years of age are highly susceptible to infection and children of age between 6 months and 1 year are the high-risk group for EBV infection.

## Introduction

The Epstein-Barr virus (EBV) is the most common herpesvirus in humans and the most common causative agent of infectious mononucleosis ^1^. It is also known as the “kissing disease” ^2^. EBV is an acute infection with a characteristic symptomatic triad of fever, sore throat and lymphadenopathy. Sprunt and Evans in 1920 coined the term infectious mononucleosis to describe an acute infectious disease accompanied by atypical large peripheral blood lymphocytes ^2^. EBV primary infection has recently risen in some countries ^3^ and children below 2 years of age are highly susceptible ^4, 5^. EBV is transmitted primarily via oral secretions and may be transmitted via penetrative sexual intercourse ^6^. Transmission may occur by the exchange of saliva among children. EBV is not spread by non-intimate contact, environmental sources, or fomites ^6^. During late adolescence 50–70 percent of teenagers get infected with infectious mononucleosis ^2^. Though it has a self-limiting course, it may sometimes lead to numerous rare, atypical and threatening manifestations. The clinical manifestations in children with EBV infection involve multiple systems and can cause severe illness, meaning that attention should be paid during diagnosis and treatment. The diagnosis of EBV infection is based on clinical features such as- fever, pharyngitis, lymphadenopathy, hepatomegaly, and splenomegaly along with leukocytosis with a predominance of lymphocytes, >10% atypical lymphocytosis, heterophile antibodies (assessed via monospot test), serum PCR for EBV DNA and serological testing including antibodies for viral capsid antigens, early antigens, and Epstein-Barr nuclear antigen.

EBV DNA PCR has high specificity and sensitivity for identifying patients with infectious mononucleosis ^7^.

## Methods

### Assessment

We retrospectively collected 253 EBV infection with serum EBV DNA positive cases from those who were symptomatically suspected as infectious mononucleosis from symptoms such as fever, pharyngitis, cervical lymphadenopathy and other lymph nodes enlargement on hospitalized patients <15 years old at Renmin Hospital, 3rd Affiliated Hospital of Hubei University of Medicine, Shiyan, (Hubei, China) during the 4-year period from January 1, 2014, to December 31, 2017. At birth, neutrophils make up around 61% of total leukocytes and lymphocytes make up around 31%. After birth, the number of neutrophils goes down and the lymphocyte number goes up, with both reaching about 45% around the 1st week of life. This process continues and by the age of 4 years, lymphocytes reaches around 50% and neutrophils reach around 42%. On growing older, the proportion of lymphocytes starts to fall and that of neutrophils start to increases. By the age of 6 years, the proportion of neutrophils reaches up to 51% and that of lymphocytes falls to 42% ^8^. Owing to this age-specific leukocytes differential, we divided patients into three age groups: <4 years, 4–<6 years and 6–<15 Years. We also made further age-specific groupings, as follows: <30 days, 1–<6 months, 6–<12 months, 1 year, 2 years, 3 years, 4 years, 5 years, 6 years, 7 years, 8 years, 9 years, 10 years, 11 years, 12 years, 13 years, and 14 years to find out the risk group for EBV infection. A diagnosis of EBV infection was achieved using real time PCR at the Pathology Department at Renmin Hospital.

### Analysis

Real-time PCR ABI iiA7 was used for quantitation of serum EBV DNA. The primers used, targeting the EBNA-1 fragment of EBV, were as follows: 5’-GTAGAAGGCCATTTTTCCAC-3’ (forward) and 5’-TTTCTACGTGACTCCTAGCC-3’ (reverse). PCR was conducted using the following thermocycling conditions: 93°C for 2 min, followed by 10 cycles of 93°C for 45 sec and 55°C for 60 sec, and then 30 cycles of 93°C for 30 sec and 55°C for 45 sec.

All data were analyzed using Microsoft Excel 2010. Age-specific prevalence was calculated. Prevalence was calculated as follows:

Prevalence = number of EBV-positive children under 15 years admitted to hospital / number of total hospital admissions for children under 15 years

## Results

**Figure 1.**
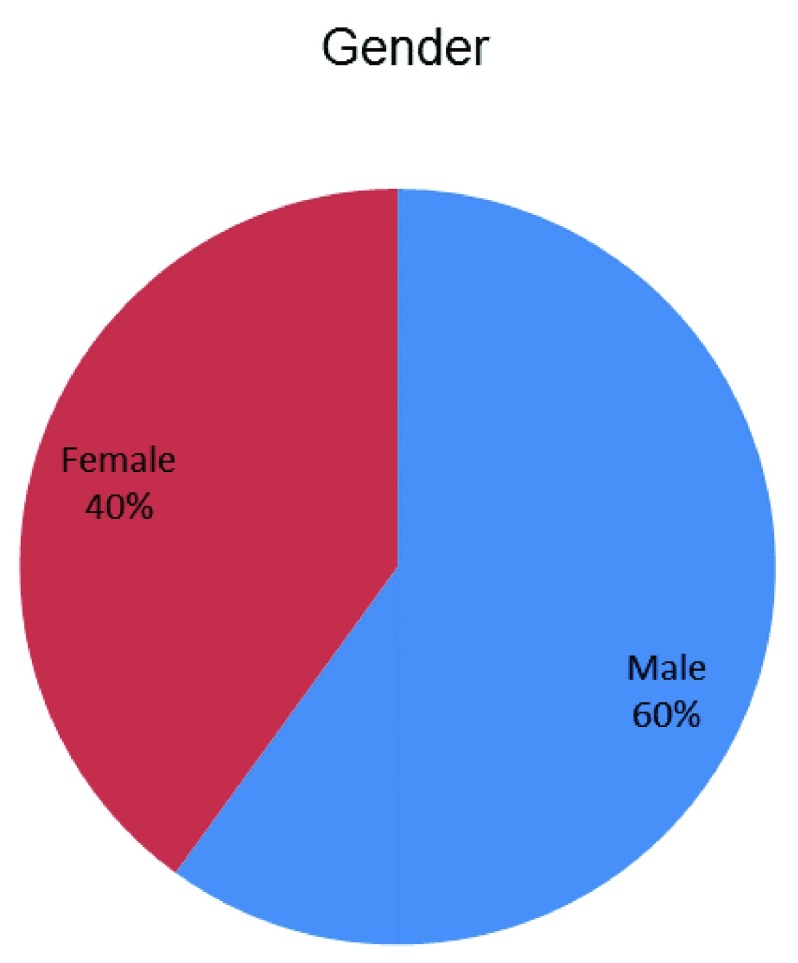
Sex distribution.

Out of the total of 253 patients, 151 (60%) were male and 102 (40%) were female. The male to female ratio was 3:2 ( Figure 1).

**Figure 2.**
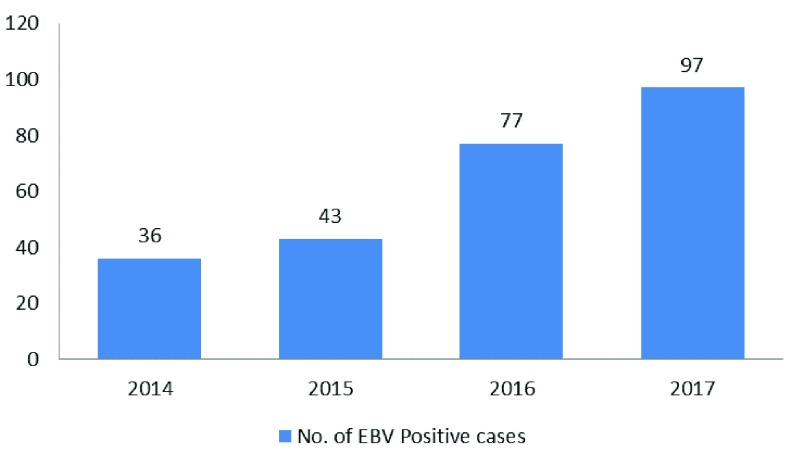
Yearly distribution of Epstein-Barr virus-positive patients.

**Figure 3.**
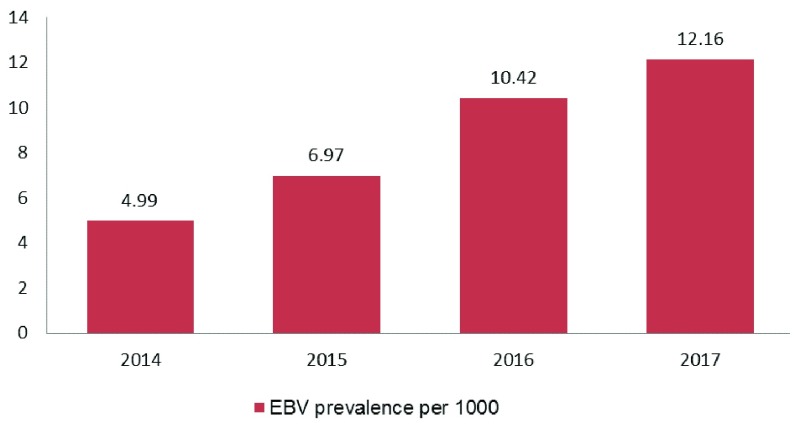
Prevalence of Epstein-Barr virus-positive patients per 1000 hospital admissions by year.

The number of serum EBV DNA-positive cases observed increased each year. There were 36 EBV DNA positive cases in 2014 (total admissions, 7202) with a prevalence of 5.00 per 1000 admissions, 43 on 2015 (total admissions, 6163) with a prevalence of 6.98 per 1000, 77 on 2016 (total admissions, 7384) with prevalence of 10.43 per 1000 and 97 on 2017 (total admissions, 7972) with prevalence of 12.17 per 1000 admissions ( Figure 2, Figure 3).

**Figure 4.**
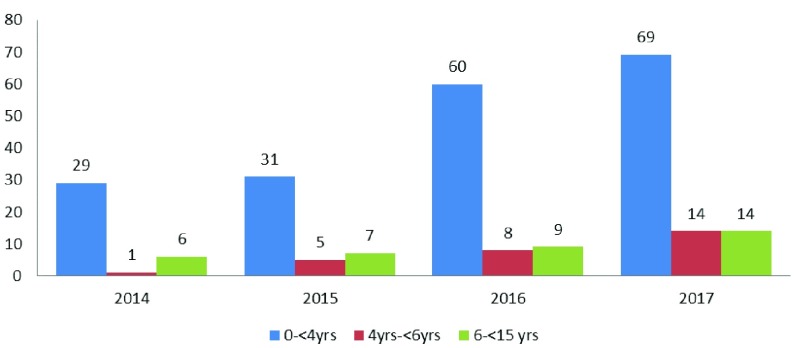
Age group distribution of Epstein-Barr virus-positive patients.

**Figure 5.**
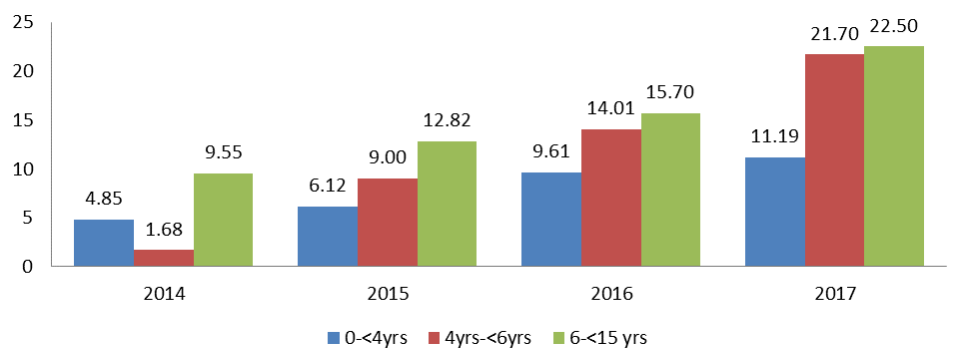
Prevalence of Epstein-Barr virus-positive patients cases of specific age groups per 1000 hospital admissions.

Over the 4 years studied here, the numbers of hospitalized children were highest in the 0 to < 4 years group. Of 253 EBV-positive patients, 189 (74.70%) were in group 0 to less than 4 years, 28 (11.06%) in the group of children aged 4 to <6 years, and 36 (14.23%) in those aged 6 to <15 years. Each year, in the group of children under 4 years the percentage of EBV positive cases were more and rate were in increasing trend ( Figure 4, Figure 5).

**Figure 6.**
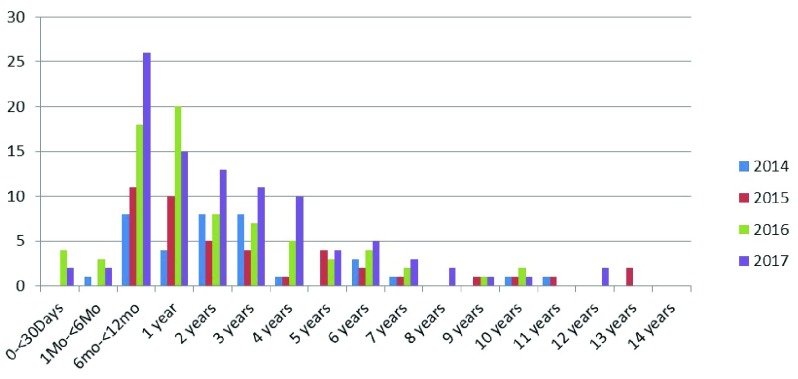
Age-specific distribution of Epstein-Barr virus-positive patients.

**Figure 7.**
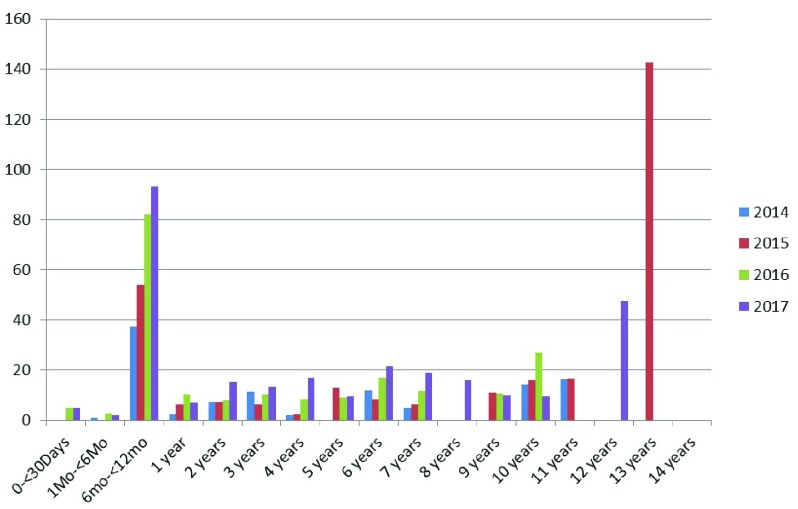
Age-specific distribution of Epstein-Barr virus-positive patients per 1000 hospital admissions.

We calculated the age-specific prevalence of EBV infection to identify the high-risk group. The number of positive cases was highest in the age group 6 months- <1 year, which decreased as age increased. Prevalence is also high in this age group ( Table 1 and Figure 6, Figure 7).

**Table 1.** Age-specific distribution and prevalence of Epstein-Barr virus.

Age	2014			2015			2016			2017		
	P	C	N	P	C	N	P	C	N	P	C	N
0–30 days	0	0	668	0	0	554	4.94	4	809	4.87	2	410
≥1–<6 months	1.05	1	954	0	0	984	2.66	3	1124	1.84	2	1083
≥6–<12 months	37.38	8	214	53.92	11	204	82.19	18	219	93.18	26	279
1 year	2.18	4	1837	6.28	10	1592	10.38	20	1925	7.00	15	2141
2 years	7.31	8	1094	7.46	5	670	8.05	8	869	15.47	13	840
3 years	11.44	8	699	6.20	4	645	10.21	7	685	13.33	11	825
4 years	1.94	1	514	2.42	1	413	8.21	5	609	16.97	10	589
5 years	0	0	343	12.90	4	310	8.90	3	337	9.63	4	415
6 years	11.95	3	251	8.16	2	245	17.09	4	234	21.73	5	230
7 years	5.12	1	195	6.36	1	157	11.76	2	170	18.86	3	159
8 years	0	0	144	0	0	127	0	0	128	15.87	2	126
9 years	0	0	92	11.11	1	90	10.75	1	93	10.10	1	99
10 years	14.28	1	70	15.87	1	63	27.02	2	74	9.52	1	105
11 years	16.39	1	61	16.66	1	60	0	0	50	0	0	55
12 years	0	0	40	0	0	26	0	0	34	47.61	2	42
13 years	0	0	19	142.85	2	14	0	0	11	0	0	24
14 years	0	0	7	0	0	9	0	0	13	0	0	12

P, Prevalence of Epstein-Barr virus (EBV)-positive cases per 1000; C, number of EBV-positive cases; N, total number of hospital admissions.

The number of total admissions and admissions of Epstein-Barr virus (EBV)-positive children under 15 years of age for each of the years 2014–2017 This dataset also contains stratifications of EBV-positive individuals by age and sex. Click here for additional data file. Copyright: © 2019 Devkota K et al. 2019 Data associated with the article are available under the terms of the Creative Commons Zero "No rights reserved" data waiver (CC0 1.0 Public domain dedication).

## Discussion

The incidence of EBV infection is higher in male children in Northern China ^9^ and Turkey ^10^. In India, the male to female ratio of EBV infection in hospitalized children is 2:1 ^11, 12^. A Korean study found the overall male-to-female ratio of EBV infection to be 1.53:1 ^13^. Our study had a male to female ratio of 3:2. During adolescence, women acquire before men the first infection by EBV ^14^. In the US EBV antibody titers were significantly higher for females ^15^.

We have found that in children under 4 years, the percentage of EBV-positive cases increased each year. However, in children aged 4–<6 years this decreased, but increased in those aged 6 to <15 years. Out of the 253 EBV positive patients, those aged under 4 years made up the highest proportion (74.7%). This drops to 11.06% in those 4–<6 years, and 14.22% in those 6–<15 years. In the study done on the Northern and Southern part of China, the seroprevalence of EBV infection is more than 50% before age 3 ^2^. Serological evidence of EBV infection is found in around 84% of Chinese children aged >9 years, with peak incidence observed at age 2–3 years ^16^. However, in a study done by Gao *et al.*, the incidence of EBV-IM peaked in children at age of 4–<6 years in Northern China ^10^. In Taiwan, the seropositive rate of EBV is high in children aged 2 years ^4^. A Danish study found that EBV infection is common in young children, and children under 3 years of age constitute the largest group of hospitalizations for acute EBV infection ^5^. In a study conducted in Poland, age of infection occurred in two peaks, i) in children aged 1 to 5 years (62%), and ii) in teenagers (24.6%) ^17^. In most developing countries nearly 70% of patients are seropositive for EBV by the age of 2 years ^18^. However in USA, the seroprevalence increased with age, ranging from 54.1% for 6–8 year-olds to 82.9% for 18–19 year-olds ^15^.

We found hospitalization for mononucleosis in all age groups. The number of positive cases was higher in the age group >6 months but <1 year, which decreases as age increases. The prevalence is also high on age group 6 month to 1 year. This indicates that the age group 6 months to less than 1 year is a high-risk group. The most common age group for hospitalization with acute EBV infection in Denmark was 1–2 years ^5^. In Asia and other developing countries most of the children are infected with EBV in early life, mostly before the age of 1 year. ^19^. According to Cocuz *et al.*, admissions for infectious mononucleosis were prevalent in young children, with most occurring in the 1–3 years age group (32.31% of the total IM Cases), followed by those 4–<6 years old (27.69% of the total IM Cases), then those 11–16 years old (26.15% of the total IM Cases) and finally those 7–10 years old (13.84% of the total IM Cases) ^20^.

 Several prior studies have reported in the last decade which shows the changes in the epidemiology of EBV infection. A Japanese study showed that the seroprevalence of EBV in 5–7 years old children was higher than 80% before the early 1990s which decreased to 59% in the years 1995 ^19^. Similarly in the USA, the study showed that the seroprevalence in 6–19 year olds declined from 72% in 2003–2004 to 65% in 2009–2010 ^21^. But, the EBV primary infection is increasing in England and Wales ^22^. Therefore, we aimed to determine the epidemiological condition of EBV infection over the last years in the Pediatrics Department of Renmin Hospital, Shiyan, China. The EBV positivity rate in hospitalized children is increasing every year. Prevalence is also increased each year. In the years 2000 to 2016, the EBV infection rate in France has increased, whereas its seroprevalence has decreased ^3^.

Although most EBV infections are self-limiting, sometimes they may lead to rare, atypical and threatening manifestations. Although serious complications during the acute phase of primary EBV infection are rare ^1^, neurological complications, like meningoencephalitis, acute encephalitis, acute cerebellitis, transverse myelitis, and myeloradiculitis, occur more frequently in children under 2 years of age ^17, 23, 24^. Furthermore, in immunocompromised individuals, there was an association observed between EBV with several tumors following reactivation of the virus from latency ^25^.

Since this study was conducted in children admitted to hospital, the results might lack generalization to the entire population, but may indicate trends and bring up questions deserving further prospective study.

Increasing primary infection of EBV in children may be due to many reasons, including that the virus is active among the population around Shiyan, airborne transmission ^26^ of the virus is higher in this area, multiple caregivers for each infant, bottle feeding, unnecessary kissing, feeding with chewed food to babies, or through hospital acquired EBV infection e.g. from health care personals, doctors or nurses. There are several reports on the intrauterine transmission of EBV, but none has been substantiated by appropriate viral studies ^27, 28^. Besides, doctors may be more familiar and experienced with the clinical presentation, symptoms, and signs of infectious mononucleosis.

The next steps should be a focus on awareness to parents and caregivers of children, and development of a vaccine against EBV to reduce the burden of EBV infection in future.

## Conclusion

The rate of hospital admission of children due to EBV infection is increasing. Children under 4 years of age are highly susceptible to infection and children of age between 6 months and 1 year are the high-risk group for EBV infection. Vaccination against EBV must be considered to reduce the burden of EBV infection in future.

## Data availability

The data referenced by this article are under copyright with the following copyright statement: Copyright: © 2019 Devkota K et al.

Data associated with the article are available under the terms of the Creative Commons Zero "No rights reserved" data waiver (CC0 1.0 Public domain dedication). 

**Dataset 1. The number of total admissions and admissions of Epstein-Barr virus (EBV)-positive children under 15 years of age for each of the years 2014–2017.** This dataset also contains stratifications of EBV-positive individuals by age and sex. DOI: https://doi.org/10.5256/f1000research.15544.d212141 ^29^.
