# Supplementary material for: Increasing Epstein-Barr virus infection in Chinese children: A single institutional based retrospective study
Source: F1000Res. 2019 Feb 15;7:1211. Originally published 2018 Aug 7. [Version 2] doi: 10.12688/f1000research.15544.2 (PMC6505636; doi:10.12688/f1000research.15544.2)
Supplement: Peer review history

# Peer review: Reviewer response for version 2

**DOI:** 10.5256/f1000research.19928.r47166

**Published:** 2019-05-07

**Authors:** Mani Prasad Gautam

**Affiliations:** 1 Department of Medicine, Bharatpur Hospital, Chitwan, Nepal; 2 National Academy of Medical Sciences, New Delhi, India

The article “Increasing Epstein-Barr virus infection in Chinese children: A single institutional based retrospective study” is a single center hospital based study which reports the rate of EBV infection in hospitalized children Although it has reported prevalence of EBV in this group, but in real meaning it is just a rate of EBV infection in hospitalized children in a hospital. Its rate might not have much meaning in general as the findings can not be generalized due to various reasons including hospital based data and also we do not have any idea about the denominator used for the calculation purpose and this denominator might differ in different hospitals and directly affects the rate of EBV reported. The main message that can be taken from this study is that the rates of EBV infection is rising in current years and its rate is higher in less than 4 years group and thereafter there is a gradual fall in its rate among older children. As authors have discussed in discussion section, we should understand that the reason for this rising trend can be different; first, real rising in EBV infection; second, increasing awareness among people and among medicos about the disease leading to frequent diagnosis of the condition. The role of other unknown factors may also have an impact.

 The study is a simple descriptive analysis, includes retrospective analysis of EBV infection from 2014 to 2017 in hospitalized children.

 The introduction section highlights history, epidemiology, clinical features and diagnosis of EBV infection. It states EBV as the most common causative agent of infectious mononucleosis and does not mention other causative agent, if there are any and it does not describe other manifestation of EBV infections. Introduction section also fails to describe the gap in the current knowledge on the subject matter, rational and need of current study. It is not clear what were the objectives of the study. 

 Methodology section describes the design of the study to some extent. It needs more on elaboration of the methodology followed, inclusion and exclusion criteria and spectrum of the pediatric subjects admitted to the hospital. The unnecessary description of peripheral blood pictures at birth and changes thereafter can be omitted. The age-specific grouping less than 4 years, 4-6 years and 6-15 years needs explanation as it seems inappropriate and equal age interval should be selected if there is no any reason to classify as chosen.

 In the result section, the word “prevalence” may not be appropriate in pertinent to this study and appropriate word should be selected. The rate of EBV infection reported is among the admitted subjects and as the spectrum of admitted subjects is unknown, it is very difficult to understand it. For example, if all the children admitted to the hospital were febrile, the EBV infection rate would have been different and if the children admitted included non-infective conditions as well, it would have been different. Unnecessary figures and double reporting can be avoided in result section. The figures 2 and 3 basically state the same, figure 2 reports in absolute numbers and figure 3 reports the rates per 1000 admissions. Similarly, figures 4 and 5 also report the same.

 Discussion section is written well. It is better to avoid the use of the word “prevalence” and make comparisons with similar studies. Limitations and suggestions for further studies should also be made and discussed. Based on the study, we can not conclude that the children under 4 years of age are susceptible to EBV infections, we can only say that the rates were higher among this group.

 Overall, the article is simple and provides few insights on EBV infection and highlights its rising trend from 2014 to 2017 with higher rate among children less than 4 years old.

I have read this submission. I believe that I have an appropriate level of expertise to confirm that it is of an acceptable scientific standard.

# Peer review: Reviewer response for version 2

**DOI:** 10.5256/f1000research.19928.r45221

**Published:** 2019-04-01

**Authors:** Michael J. Goldacre

**Affiliations:** 1 UK Medical Careers Research Group, Unit of Health-Care Epidemiology, Nuffield Department of Population Health, University of Oxford, Oxford, UK

It is hard to assess what the findings truly mean, because the study population is not a representative sample of the general child population of the area studied. It is a study of children admitted to hospital with clinical findings suggestive of EBV infection who are shown to have had the infection (the ‘numerator’), expressed as a percentage of all children of the same age admitted to the hospital (the ‘denominator’). It is therefore not true prevalence, but the prevalence in hospitalised children. The numerator is potentially affected by factors that influence the referral of children to hospital and the clinical decisions to admit them. The denominator cannot be said to be representative of the general population of children. Changes over time in the prevalence rate could be affected by changes to the numerator, which is what the authors have assumed (i.e. more children affected by EBV); or by changes in the referral or admission practices in respect of children with symptoms suggestive of EBV infection; or they could be affected by changes to the denominator population. To illustrate the latter point, an increase in admissions of children with non-infective diagnoses, eg injuries, would tend to reduce the rate of children with EBV per 100 children admitted overall.

 It is also impossible to know how to interpret how the authors’ data on ‘prevalence’ compare with those reported by other studies, because the sampled populations may be quite different. For example, reference 21 reports a study of people in the National Health and Nutrition Examination Surveys (NHANES) which comprise a representative sample of the US population (and which may therefore be quite different from a hospitalised population).

 The authors cannot do anything about the nature of the subjects studied by them; but they can describe and discuss this as a potential limitation of the study.

I have read this submission. I believe that I have an appropriate level of expertise to confirm that it is of an acceptable scientific standard, however I have significant reservations, as outlined above.

# Peer review: Reviewer response for version 2

**DOI:** 10.5256/f1000research.19928.r44512

**Published:** 2019-02-22

**Authors:** Anna Mania

**Affiliations:** 1 Department of Infectious Diseases and Child Neurology, Karol Marcinkowski University of Medical Sciences, Poznań, Poland

Suggested amendments were done appropriately. I have no further comments

I have read this submission. I believe that I have an appropriate level of expertise to confirm that it is of an acceptable scientific standard.

# Peer review: Reviewer response for version 1

**DOI:** 10.5256/f1000research.16956.r37372

**Published:** 2018-09-12

**Authors:** Anna Mania

**Affiliations:** 1 Department of Infectious Diseases and Child Neurology, Karol Marcinkowski University of Medical Sciences, Poznań, Poland

The study presents results of retrospective analysis conducted to estimate the overall prevalence of EBV infection and identify high risk groups among children.

 This study included 253 patients under 15 years of age positive for EBV DNA by PCR, were admitted to the Pediatrics Department in China during a 4-year period from 2014 to 2017. Patients were divided into three groups; 0-<4years, 4-<6years and 6-<15years. The percentage and prevalence of EBV DNA-positive cases was calculated on that basis.

 The highest rate of EBV infection (74.7%) was observed in the group under 4 years of age, 11.06% and 14.22% in the 4-6 years and in the 6-15 years group, respectively.

 The authors mention increasing number of EBV-infected individuals in recent years. Noticing the highest number of cases in children between 6 month to 1 year.

 Certain limitations of the study were visible:

1. The authors confirm EBV infection by PCR, however I could not find the information concerning the type of the specimen – blood, saliva, urine, anything else?
2. Significant proportion of EBV infected patient may be asymptomatic; the term infection is not equivalent to the disease, therefore it is a pity that data concerning clinical symptoms are not mentioned.

Minor comments:

1. Citing articles are not equivalent to given numbers e.g –study conducted in Poland is given at the 25 positions in the list, not 18;

I have read this submission. I believe that I have an appropriate level of expertise to state that I do not consider it to be of an acceptable scientific standard, for reasons outlined above.

# Response

**Published:** 2019-02-04

**Authors:** Kiran Devkota

**Affiliations:** Hubei University of Medicine, China

Respected Professor

 Thank you so much for you time and consideration. I have tried to make clear on the queries that you have noted.
